# Barriers to Participate in Support Groups for People Living with HIV: A Qualitative Study with Men Receiving Antiretroviral Treatment in a HIV Clinic in Mthatha, South Africa

**DOI:** 10.5539/gjhs.v4n6p119

**Published:** 2012-09-18

**Authors:** Sphiwe Madiba, Vuyokazi Canti-Sigaqa

**Affiliations:** 1 Department of Environmental and Occupational Health, School of Public health, University of Limpopo, Medunsa Campus, South Africa

**Keywords:** disclosure of HIV status, support groups, HIV/AIDS, South Africa, participation, antiretroviral therapy

## Abstract

Support groups are the most common and popular way of providing social support for people living with HIV and AIDS (PLWHI). Nevertheless, HIV positive men are reluctant to attend support groups, and in most mixed gender support groups, women outnumber men. The study used a sample of men accessing antiretroviral treatment (ART) from a HIV clinic in South Africa, to examine their perceptions of support groups and explore their reasons for nonparticipation in such groups. Five focus groups interviews were conducted with 50 HIV positive men. Their age ranged from 28-70 years, all had disclosed their HIV status to partners and family members and were receiving ART for more than a year. The main barriers for nonparticipation related to issues on support groups were; Unavailability of support groups in local communities including; no access, the timing of meetings and lack of transport money. Fear of unintended disclosure of HIV status due to breach of confidentiality with resulting stigma and social rejection. On a personal level, participants felt that they had adequate support at home. Participants would consider participating if men only support groups are initiated, support groups are held on weekends, and they are provided with more information on support groups. Health care providers have a critical role to play in creating awareness of and education on the role of support groups for PLWHI. Support group planners should consider men only support groups which has been shown to have positive outcomes and facilitates member participation.

## 1. Introduction

Support groups were proposed as a key psychosocial intervention for people living with HIV and AIDS (PLWHI) since the beginning of the HIV epidemic. Countries like Uganda, Zambia, and Zimbabwe were in the forefront for the establishment of community based care and support for PLWHI in Sub Saharan Africa (Russell & Schneider, 2000). A key element in care and support is the provision of psychosocial support through the establishment of peer support groups for PLWHI. Psychosocial support addresses the on-going psychological and social problems of PLWHI, their partners, families, and caregivers (World Health Organisation [WHO], 2004). In many parts of the world, HIV support groups were established as an integral part of care and support for PLWHI by community based HIV organisations, governmental organisations, and by PLWHI (S. C. Kalichman & Sikkema, 1996; Liamputtong, Haritavorn, & Kiatying-Angsulee, 2009; Russell & Schneider, 2000; Shippy & Karpiak, 2005). As a result, support groups have been used as a key intervention to help PLWHI in dealing with the changes that come with their illness for the past three decades (Spirig, 1998). Furthermore, HIV support groups have become the most common and popular way of providing social support services for PLWHI in resource-limited settings (Kalichman & Sikkema, 1996; Visser & Mundell, 2008).

The purpose of support groups amongst others is to help members cope with stressful events, neutralize stigma and allow members to practice new behaviors (Kalichman & Sikkema, 1996; Visser & Mundell, 2008; Wood, 2007). A systematic review of literature shows that support groups are a useful, effective, helpful, and supportive intervention for PLWHI (Spirig, 1998). Participating in support groups enhances the quality of life, decrease isolation and feelings of shame, improve self-care behaviors, and create mutually empathetic relationships among members (Lennon-Dearing, 2008). The interaction with other HIV positive people is an important component in the success of the support groups and creates a sense of belonging (VanDevanter et al., 2000; Visser & Mundell, 2008). Furthermore, participation in support groups offer benefits of improved medication compliance, decreased risk behavior for re-exposure, and reduced feelings of shame (Lennon-Dearing, 2008). An additional benefit is that support groups can play an important role in addressing HIV prevention behaviors such as safer sex practices, HIV status disclosure, and condom use to decrease the spread of the disease (Kalichman, Rompa, & Cage, 2005).

Although support groups for PLWHI are meant to cater for the needs of men and women, data from several studies show that HIV positive men are reluctant to attend support groups. Consequently, in most mixed gender HIV support groups, women outnumber men (Chazan, 2006; Shacham et al., 2008; VanDevanter et al., 2000; Visser & Mundell, 2008). One of the reasons for low participation of men in support groups is the reluctance to publicly disclose their HIV status. Mundell et al. (2011) argue that disclosure of HIV status is inevitable in HIV support groups, and people who are not ready to disclose may fear participation for this reason. Furthermore, people may also fear the possibly resulting stigma and discrimination. Data from mixed gender support groups show that access and availability of support groups in local communities are related to barriers for participation in support groups. Issues of money for transport to and from meetings, work, and lack of time to attend meetings, were reported as barriers to participate in support groups (Liamputtong et al., 2009; Morrow et al., 2001; Visser et al., 2005). Other reasons for nonparticipation in support groups are; the large size of support groups, concerns about confidentiality, presence of existing family support, and personal disinclination (Heyer, Mabuza, Couper, & Ogunbanjo, 2010; Liamputtong et al., 2009). Furthermore, it has been reported that men living with HIV/AIDS suffer in silence to protect their life, and are less likely to seek health care (Maboshe, 2008).

The poor participation of men in support groups has been observed in many health care facilities providing HIV care and support to PLWHI in South Africa. This is despite support groups being the commonly advocated psychosocial support intervention for PLWHI. The lack of participation of PLWHI in support groups is a major concern for health care providers, given the increasing numbers of people who seek HIV testing and counselling in South Africa. Testing for HIV is the first step in the reduction of HIV transmission, and support groups assist PLWHI to obtain HIV-related information to develop strategies to change behavior. Understanding the barriers to participate in support groups will inform the development of interventions to increase men’s participation in HIV prevention programs. However, there are limited studies describing the profiles and barriers to participation in support groups among PLWHI. This study was conducted to address the lack of information about HIV positive men and their participation in support groups. The aim of the study is to examine HIV positive men’s perceptions of HIV support groups and explore their reasons for nonparticipation in such groups.

## 2. Methods and Materials

### 2.1 Study Design

We conducted focus group (FG) interviews with HIV positive men receiving ART from the Infectious Disease Clinic (IDC) of Mthatha Hospital Complex in KSD Municipality, in the Eastern Cape Province, in South Africa. The IDC provides HIV care and support for PLWHI from about 10 rural villages and 8 urban townships in and around the KSD Municipality. Data were collected between September and November 2009. At the time of data collection, there were about 500 PLWHI enrolled for ART in the IDC.

### 2.2 Data Collection

FG interviews were conducted by the second author trained in facilitating focus groups. A research assistant also trained in qualitative methods assisted with recruitment and moderation of the FGs. An open ended FG guide was used for the FG interviews. The guide was developed in English and translated into Xhosa, a local language spoken by participants in the study setting. Participants were recruited during their routine monthly visit to the clinic. Following an information sharing session, participants who were interested in being a part of the study were asked for their contact details. Appointments were then scheduled for the FG interviews at times convenient to the participants. All participants chose the days that they were collecting their ART medication as suitable dates for the FG interviews. Participants were selected if they were HIV positive men aged 18 years and above, were receiving ART, were not members of any support group, and volunteered to participate in the FG interviews. Participants who were very ill were excluded from participating.

The research assistant phoned the participants to remind them about the schedule FG appointment two weeks and then two days before the FG interviews. On the scheduled day for the FGs, we obtained a written informed consent from individual participants prior to the start of the interviews. The FG interviews were conducted in Xhosa and were audio record with the participants’ permission. The interviews lasted for 60 to 90 minutes, and participants were served refreshments at the end of the interviews. Five FG interviews were conducted, and each FG interview had an average of ten participants with a total of 50 participants.

At the end of the FG interviews, participants were asked to fill a brief self-administered tool. The questionnaire collected demographic information including their age, level of education, marital status, employment status, date of HIV diagnosis, and period on ART. The tool was translated into Xhosa and the researchers assisted participants who could not read and write to fill the questionnaire.

The Medunsa Research Ethics Committee of the University of Limpopo granted ethical approval for the study. Permission to conduct the study was obtained from the Eastern Cape Provincial Department of Health, and the Chief Executive Officer of Mthatha Hospital Complex. Participation in the study was voluntary, and an informed consent was obtained from the participants prior to the FG interviews.

### 2.3 Data Analysis

The primary coding of the transcripts was undertaken by the authors. Transcripts were transcribed in Xhosa and translated into English by the second author who reviewed transcripts for accuracy by replaying each interview recording whilst reading and translating the transcripts. Both authors are well conversant with English and Xhosa and verified that the translations were a true reflection of the interview. Multiple readings of one transcript were undertaken by the authors who independently identified major themes related to participation in HIV support groups. A code list was subsequently developed and reviewed for consensus on the definitions of the themes and sub themes. The transcripts were recoded if a new code emerged or an existing code was revised. NVivo version 8 was used in the application of themes to the remaining transcripts. Themes that were consistent in terms of the process of disclosure became categories.

Various strategies were employed to ensure trustworthiness; we conducted the FG interviews in the local language, obtained detailed field and interview notes, recorded the FG interviews, transcribed transcripts verbatim in Xhosa, held peer debriefing sessions after each FG interview to discuss emerging themes, verified raw data during translation, used a computer software for analysis, and employed researcher triangulation during data analysis (Creswell, 2007; Patton, 2002).

## 3. Results

### 3.1 Sample Description

Fifty HIV positive men participated in five FG interviews, their age ranged from 28-70 years, 29 (58%) were single, 15 (30%) were married, 4 (8%) were widowed, and 2 (4%0 were divorced. Most 38 (75%) were unemployed, 26 (52%) had secondary education, 18 (36%) had primary education and 6 (12%) had grade 12 education. Most of the participants 36 (72%) were living with HIV for 1-5 years, 11 (22%) for 6-10 years and 3 (6%) were living with HIV for more than 10 years. With regard to time on ART, 40 (80%) have been on ART for 1-5 years, only 1 (2%) participant was on ART for 6 years and 9 (18%) for less than a year.

### 3.2 Themes

Five major themes related to participation in support groups for PLWHI emerged from the data analysis; awareness of support groups, perceptions of support groups, barriers for participation, and intention to attend. Sub themes for barriers for participation in support groups included access to support groups, location of support groups, unintended disclosure, timing for meetings; men only support groups, support from family. Each of these themes is described below.

#### 3.2.1 Awareness of support groups

Participants responded to a question on their awareness of support groups for people living with HIV. The data show that some of the participants were not aware of support groups in their communities and in the clinic where they collected their ART medication.

*Where I am staying, there are no support groups. I have little knowledge that they discuss things like AIDS (39 year old male)*.

*The reason why we do not know about support groups is because when we are here at the clinic, nobody gives us information about them, and that is the reason why I do not participate in them (33 year old male)*.

#### 3.2.2 Perceptions of support groups

Though participants were not members of any support group during field work, they were asked about their views about support groups. The data show that participants were positive about support groups.

*I personally see them as helpful because you are able to share your experiences and how to help each other. Even now if you regularly attend support groups, you are able to assist another person (28 year old male)*.

*I usually hear when people speak about them, saying they are very good, and that people are helping each other there. I could be a member if one could be established in my community, it could be a great help (28 year old male)*.

*In the support groups, is where HIV positive people who have not yet started with ARVs get advice on how to live positively. The people who attend support groups look well even though they have not yet started with the treatment (47 year old male)*.

In contrast, some of the participants had negative perceptions about support groups, citing various reasons for their attitudes.

*The participants in the support groups are not trustworthy, I would not tell everyone about my status, I would only disclose to those that I trust (46 year old male)*.

*I can say that support groups are helpful, but sometimes they are not. If your condition is worse, you get inspired when you learn from other member’s experiences and challenges. But because of stigma, everything that is done destroys you as an HIV positive person. Most people still have a negative attitude towards people who are living with HIV because of stigma. Secondly; even your confidence is being destroyed by the community’s negative attitude. I do not blame people who are not part of support groups if the community still stigmatizes HIV positive people (32 year old male)*.

#### 3.2.3 Barriers for Participation

A recurring theme throughout discussions with men revealed that there were many barriers to attend support groups.

3.2.3.1 Access to Support Groups

Most participants had not heard about support groups before the focus group interviews. They consequently said that the unavailability of support groups in their local communities was a reason for not attending.

*In my community, there are no support groups…, I would like to join, another stumbling block is my work, I have very little time, but if they could be established where I stay I would attend*(*36 year old male*).

*Support groups are far from where I stay…, distance is a problem for me, as well as the times when support group meetings start (44 year old male)*.

3.2.3.2 Location of Support Groups

The location where the support group meetings are held was a barrier for some participants. Data show that to some participants the issue of the venue where the support groups are run is linked to the fear of being recognised as an HIV positive person.

*I like support groups, but I have a problem with the place where they are run, for instance in my local clinic. If they can be run somewhere else, I can participate in the support groups (50 year old male)*.

*My reason not to be interested to take part in a support group that is run in my community, is because people are making fun of us when they know that you are infected with HIV. It might be better to attend in an area where you do not stay. Then I could go and join if it is at a distance from where I stay (31 year old male)*.

3.2.3.3 Unintended Disclosure

For several participants, the reason for not attending support groups was the fear of being recognised as a HIV infected person. They perceived attending support groups as comparable to disclosing their HIV status to other people.

*I would not say I will join the support group in my community because I do not want people to known that I am positive; I do not want to disclose my status. I would rather join a support group that is established here in this clinic (50 year old male)*.

*My problem is that I do not want to expose myself…, I do not want other people to know that I am HIV positive (46 year old male)*.

*The people just despise you once they are aware that you are infected with HIV. This is the reason that I will not participate in the support groups. Some other people mock you because of your status, especially if the support group is in the community where you live (41 year old male)*.

3.2.3.4 Timing for Meeting

The time scheduled for support group meetings was also mentioned as a reason for not attending support groups. Timing mostly affected employed participants.

*I think support groups are good, but I do not have time to attend because I am working. Time is a problem that is why I am unable to participate (42 year old male)*.

*I wanted to attend, but my employer does not want to release me, so I have no time (40 year old male)*.

3.2.4 Men only Support Groups

Mixed gender support groups were one of the reasons for not participating. Participants strongly advocated for men only support groups and cited various reasons for that.

*There are things that men might not feel free to share amongst women. I think men could advise each other freely without the presence of women. Secondly, women like to dominate in meetings especially when they are together with men. If men are alone, they will be responsible for their own group (43 year old male)*.

*Most men feel uncomfortable amongst women. I personally feel uncomfortable as well. I would like that we are separated from women, they should have their own groups and we our own. Then I think I can be able to attend (31 year old male)*.

3.2.5 Support from Family

One other reason for not attending support group meetings was because some participants felt that they received adequate support from their families and partners.

*My support group is my wife and kids, they look after me, and they give me support (46 year old male)*.

*I think I receive good support at home with my family. Another thing I am very busy, I will not be able to join any support group. For most of the time, I receive the support that I need at home (29 year old male)*.

3.2.6 Intention to Attend

Several participants would like to participate in support groups in the future. They further suggested conditions that would facilitate their participation.

*I would like to join, if they could be established where I stay I would attend. I do not mind people knowing how I am, but because they are not there, otherwise I would not mind attending and joining them (36 year old male)*.

*My suggestion is that it could be better if the support group meeting could be on the same day we fetch our medication (35 year old male*).

*If it is possible, I will like the times for meetings to be adjusted so that they do not clash with work. I wish these meetings could be held over the week end, and not during the week (28 year old male)*.

In contrast, a few participants had no intention to attend support groups and cited various reasons for their view point.

*I have a slight problem about the support groups…, I think that I will not gain anything more than what I received from the counselling. I think it would be better if someone who has an experience about what happens in support groups could share his knowledge with us. Share things that are different from what we heard during the counselling process. Maybe I could be interested (28 year old male)*.

*I do not see a need for me to attend the support groups because I was counseled and that was enough for me. I do not see any need now for further counseling (33 year old male)*.

*I do not want to pre occupy my mind with my positive status. Even if these groups were available in my area, I was not going to attend. I am also busy…, I am studying* (*35 year old male)*.

## 4. Discussion

The study explored the views of HIV positive men about participation in support groups. Most of the participants learned about their HIV status after a long history of illness, and were encouraged by their partners or families to go for HIV test. Disclosure of the HIV status for most occurred immediately after diagnosis. In a number of cases, the participants were informed about their HIV positive results in the presence of their partners or family members. Because participants were critically ill, most were accompanied to the health facility for the HIV test by partners or family members. Participants who were not accompanied for the HIV test, also disclosed to partners and families shortly after diagnosis because they needed to have treatment partners to enrol in the ART program. In South Africa, having a treatment partner is a requirement for enrolment in the ART program (National Department of Health [NDoH], 2004). Similar patterns of HIV testing were reported in western Kenya, where many patients seek HIV counseling and testing due to medical complications following a long history of illness (Shacham et al., 2008).

Data show that most participants were aware about HIV support groups in general, although a few did not have any idea about the HIV support groups that were run in the ART health facility where they collect their monthly treatment. This was despite most (80%) receiving ART for more than a year (range 1-5 years), and the fact that support groups are running four times a week in the study facility. One of the reasons for the poor awareness of support groups might be because most participants tested for HIV at a time when they were critically ill and did not remember what was said during counselling. The findings further suggest that HIV positive people do not necessarily receive on-going counselling as part of their treatment and support. It is during on-going counselling sessions that participants are informed about HIV support groups. Findings from a hospital based support group show that the reasons participants attended for the first time were because they were referred to the support group by a healthcare worker. Most continued attending the support group after gaining information about HIV (Heyer et al., 2010). The data further show that participants, who knew about HIV support groups in this study, did not have an in-depth understanding of the how, the where, and the why of support groups. Consequently, most could not articulate their views on the perceived benefits of support groups. However, participants who were aware of support groups perceived support groups as valuable to PLWHI. These participants had a positive attitude towards support groups and were of the opinion that PLWHI should attend support groups.

Almost all the participants never participated in support groups, and they cited various reasons for their nonparticipation. Our finding is in line with other studies showing that participants who had never attended support groups reported more perceived barriers to participation than participants who had previously attended support groups (Walch, Roetzer, & Minnett, 2006). One of the barriers to participate in support groups in this study was because support groups were not available in the communities where the participants resided. The unavailability of support groups in the local communities could be one of the reasons that participants lacked awareness and in-depth understanding of support groups. Distance to support group meetings was also identified as a barrier to participate; most of the unemployed participants could not afford transport to and from support group meetings. Finances, time for support group sessions, and distance to support group meetings were previously mentioned as barriers to participate in support groups (Morrow, Costello, & Boland, 2001).

In contrast, some of the participants preferred not to attend support groups in their local communities for fear of being recognised as being HIV positive. Participants perceived support groups as lacking confidentiality, and were of the opinion that their status would be known in their communities, and they would be stigmatized and socially rejected. Stigma remains a major concern within the health care services field across societies worldwide. It has been documented that stigma levels are elevated in low-resource areas of the world among people who have lower levels of knowledge about HIV (Liu et al., 2006; Mak, Poon, Pun, & Cheung, 2007). It has also been documented that participation in support groups assists PLWHI to deal with stigma and isolation, provides emotional support, improves HIV knowledge and promotes positive living. It is therefore, assumed that the level of HIV knowledge among participants in this study is low, given their nonparticipation in support groups. Similar to findings from previous studies, participants associated participating in support groups in their communities, to disclosing their HIV status to other people they would prefer not to know (Mundell et al., 2011). It should also be noted that none of the participants in this study disclosed their HIV status to people outside of their close family and partners, and because disclosure in support groups is inevitable, participants preferred not to participate in local support groups. Heyer et al. (2010) also found that participants in their study were concerned about confidentiality because of the large size of their support groups. Issues of HIV related stigma and fear of participation in support groups were previously reported in other studies (Liamputtong et al., 2009; Morrow et al., 2001).

In addition, the fear of stigma influenced the intentions for future participation in support groups. According to Gilbert & Walker (2010), perceived stigma remains a significant barrier that limits the potential value of HIV treatment care and support. Participants in this study preferred to join support groups in communities where they were unknown to the members of the community. Some felt that the clinic where they collect their monthly ART medication would be an ideal place for support group meetings. Because of stigma and discrimination, most HIV positive people prefer to receive HIV treatment, care and support from health facilities far from their communities. PLWHI believe that when they attend the local clinic for HIV care they would be recognised, and their HIV status would be made known to other people in the community. Therefore, the preference for the clinic as an ideal venue is because most participants were receiving their ART from clinics far from where they reside.

The timing of the support group meetings was not convenient for both the employed and the unemployed participants. Employed participants lacked the time to attend support groups because meetings are held during week days. They reported not being able to take time off to attend support groups meetings because they are given only one day to collect ART medication. The unemployed participants lacked transport money to attend support groups because for some, the support group meetings do not coincide with the clinic days for collection of ART medication. Time was found to be a barrier to attending support group meetings in previous studies (Liamputtong et al., 2009; Morrow et al., 2001; Visser & Mundell, 2008). On the other hand, for participants who lived far from the clinic, even when the support group meetings coincided with clinic days because they travelled far they were unable to attend the meetings.

Attending support groups with women was one of the key barriers to participate. Participants felt strongly about having men only support groups. Most advocated for men only support groups because they were uncomfortable to attend with women. They perceived women as talking too much, unable to keep secrets, and dominating support group meetings when they attend with men. Participants felt that it would be easy for them to discuss sexual issues as men only. They further advocated for male facilitators of support groups. Although gender based HIV support groups are not common in South Africa, Lennon-Dearing (2008) argues that women only HIV support groups provide members with role models of women living with HIV that normalize and destigmatize members’ situations. The author further argues that gender homogeneity in the composition of support groups may play a significant part in the outcome of HIV support group participation and is an important consideration for support group planners. Wood (2007) also argues that homogeneity is a critical component of successful support groups for males.

One other reason for nonparticipation was lack of information about support groups for PLWHI. Participants reported that they were not informed about support groups running in the clinic or in their communities. The clinic was also not the preferred venue for support groups because participants perceived the clinic as lacking confidentiality. Heyer et al. (2010) found that the long queues at the HIV clinic and negative staff attitudes towards participants were barriers for participation in support groups.

For some participants, the reasons for not attending support groups were personal and not related to any of the barriers discussed above. Similar findings were previously reported (Lyttleton, 2004). The pre and post-test counselling offered when participants had their HIV test, was perceived by some participants as adequate, resulting in no further need for counseling or participation in support groups. Participants were not convinced that they would learn anything different from support groups to what they learned during post-test counselling. While other participants perceived the support they received from partners and family members to be sufficient. Family support was a reason for not joining HIV support groups in other studies (Lyttleton, 2004). Support networks are crucial elements in the lives of PLWHI, with informal social networks, consisting of family and friends, playing an important role. Emotional, informational, and instrumental assistance received from family and friends can lessen the negative effects of stigmatization, isolation, and depression (Emlet 2006; Shippy & Karpiak, 2005). All the participants in this study disclosed to family members and partners when they tested HIV positive. Data show a close association between disclosure and perceived social support, and that PLWHI who disclose to family enjoy greater social support that may buffer their emotional distress (Emlet, 2006; Kalichman, DiMarco, Austin, Luke, & DiFonzo, 2003; Serovich, Brucker, & Kimberly, 2000). More social support is also associated with fewer negative effects of stigma and better outcome regarding medical adherence (Shippy & Karpiak, 2005). In addition, participants in this study were of the opinion that support groups were only for people who cannot cope with their HIV status. Similar findings were previous reported (Kalichman & Sikkema, 1996).

Selection bias may limit the generalizability of the study findings. This study involved collecting data from HIV positive men who were not members of HIV support groups. The views of men who were members of support groups are not reflected here. Nevertheless, socio demographic characteristics were used in selecting participants to ensure that the selected participants cut across the age groups of men accessing ART medication in the study facility.

## 5. Conclusion

Although it is acceptable and expected that not everyone with HIV infection join support groups, the low participation in support groups in this study, is of concern. Data from several studies show that attending support groups is associated with positive changes in emotional, behavioral and physical health of participants. The study concludes that because of nonparticipation in support groups, participants are not benefiting from the emotional and informational support gained through participation in support groups. This further explains the heightened level of fear of stigma experienced by the participants.

Understanding barriers to participate in HIV support groups may assist support group planners to develop responsive interventions for PLWHI. Although participants cited a number of barriers for not participation in support groups, they also suggested what would facilitate their participation. Support group planners should consider men only support groups; given that homogeneity in the composition of groups has been shown to have positive outcomes and facilitates member participation. Health care providers have a critical role to play in creating awareness of and education on the role of support groups for PLWHI.
